# Therapeutic effect of integrin-linked kinase gene-modified bone marrow-derived mesenchymal stem cells for streptozotocin-induced diabetic cystopathy in a rat model

**DOI:** 10.1186/s13287-020-01795-4

**Published:** 2020-07-10

**Authors:** Yi Huang, Jie Gao, Yiduo Zhou, Shuo Wu, Yunpeng Shao, Haoliang Xue, Baixin Shen, Liucheng Ding, Zhongqing Wei

**Affiliations:** 1 Department of Urology, Nanjing Medical University Second Affiliated Hospital, No.121 Jiangjiayuan Road, Gulou District, Nanjing, 21000 China; 2 Department of Urology, Affiliated Hospital, Jiangnan University, Wuxi, China; 3 Department of Urology, Jiangdu People’s Hospital of Yangzhou, Yangzhou, China

**Keywords:** Bone marrow-derived mesenchymal stem cells, Diabetic cystopathy, Integrin-linked kinase, Angiogenesis, Proliferation, Antiapoptosis

## Abstract

**Background:**

Diabetic cystopathy (DCP) is a chronic complication of diabetes mainly within the submucosal and muscular layers of the bladder due to the hyperglycemia-induced ischemia. As no effective therapies are currently available, the administration of optimized mesenchymal stem cells (MSCs) provides a potential treatment of DCP. Thus far, new strategy, such as genetic modification of MSCs, has been developed and has shown promising outcomes of various disorders.

**Methods:**

This study was conducted using integrin-linked kinase (ILK) gene-modified bone marrow-derived stem cells (BMSCs) for streptozotocin (STZ)-induced diabetic cystopathy in a rat model. In total, 68 male Sprague-Dawley rats were randomized into five groups: sham control (control group, *n* = 10); DCP model alone (DM group, *n* = 10); DCP rats intravenously treated with BMSCs (BMSC group, *n* = 16); DCP rats accepted adenoviral vector-infected BMSCs (Ad-null-BMSC group, *n* = 16) and DCP rats accepted ILK adenoviral vector-infected BMSCs (Ad-ILK-BMSC group, *n* = 16). Diabetic rats accepted cell transplantation in the experimental group (2 rats per group) were sacrificed for the bladder tissue on the third day, 7th day, and 14th day of treatment respectively ahead of schedule. At 4 weeks after treatment, all rats in five groups accepted urodynamic studies to evaluate bladder function and were sacrificed for bladder tissue.

**Results:**

Our data showed that the underactive bladder function was significantly improved in DCP rats intravenously treated with ILK gene-modified BMSCs compared to those in the DM, BMSCs, and Ad-null-BMSC group. Meanwhile, we found that gene-modified BMSC treatment significantly promoted the activation of the AKT/GSK-3β pathway by increasing phosphorylation and led to the enhancement of survival. In addition, the expression levels of angiogenesis-related protein vascular endothelial growth factor (VEGF), basic fibroblast growth factor (bFGF), and stromal cell-derived factor-1 (SDF-1) were significantly higher in the Ad-ILK-BMSC group than that in the DM, BMSCs, and Ad-null-BMSC group as assessed by enzyme-linked immunosorbent assay and western blot. As two indicators of vascular endothelial cell markers, the expression of von Willebrand factor (vWF) and CD31 by western blot and immunofluorescent staining revealed that the percentage of the vascular area of the bladder tissue significantly increased in Ad-ILK-BMSC group compared with the BMSCs and Ad-null-BMSC group on the 14th day of treatment. Histological and immunohistochemical staining (hematoxylin and eosin (HE), vWF, Ki67, and TUNNEL) on the bladder tissue revealed statistically different results between groups.

**Conclusion:**

ILK gene-modified BMSCs restored the bladder function and histological construction via promoting the process of angiogenesis and protecting cells from high glucose-associated apoptosis in STZ-induced DCP rat model, which provides a potential for the treatment of patients with DCP.

## Background

Diabetic cystopathy (DCP) is likely the most common complication affecting well over 50% of diabetic individuals and a main cause of urinary tract infection in the world. Lee et al. estimated that the prevalence rate of urodynamically diagnosed DCP ranged from 25 to 90% in the diabetic population [1]. The term “DCP” was first used by Frimodt in 1976 to describe increased bladder capacity and post-voiding residual volumes in diabetic patients, accompanied by decreased bladder sensitivity and contractility [2]. At present, DCP treatment has mostly focused on reducing infection and hydronephrosis from uroschesis in the chronic phase. Although medicine and surgical therapies have provided promising results in clinical practice, long-term follow-up results have shown that these treatment options are unreliable with obvious adverse effects and also create a higher long-term socioeconomic burden [3].

The main pathology of DCP is an impaired endothelium due to ischemia in response to hyperglycemia which triggers a cascade of biochemical intracellular remodeling signaling processes [4–6]. However, contrary to our current concept about DCP, some studies have revealed that hyperglycemia does not result in a loss of parasympathetic sensory nerve fibers in the bladder [7]. Therefore, around the world, there has been considerable interest in improving underactive bladder associated with DCP via bladder blood flow [8].

In recent years, increasing evidences have shown that stem cell-based therapy is emerging as a viable restorative treatment for DCP [9, 10]. In particular, mesenchymal stem cells (MSCs) have been commonly used in the animal models and clinical trials for two decades, and shown a promise in the treatment of numerous diseases, mainly tissue injury and immune disorders [11]. Moreover, intensive studies have been conducted to demonstrate that due to their capacity of secreting multiple angiogenic and growth factors, MSCs have a great potential in the repair of damaged vascular and parenchymal cells, benefiting tissue regeneration and function recovery of ischemia diseases [12]. MSCs can be derived from several tissues including the umbilical cord blood, adipose tissue, and bone marrow. As two main autologous MSC types, adipose-derived mesenchymal stem cells (ADSCs), and bone marrow-derived mesenchymal stem cells (BMSCs) are generally applied in tissue regeneration investigations due to their abundance and easy access. And some previous studies have found that the source of MSCs (bone marrow vs adipose tissue) has no impact on the regeneration of tissue-engineered urinary bladder [13]. According to recent literatures, BMSCs have been proved to undergo directed differentiation toward endodermal derived urothelium and develop into mature bladder tissue in the appropriate signaling environment [14]. Moreover, BMSCs have also been reported to inhibit hypoxia-induced inflammatory and fibrotic pathways in bladder smooth muscle cells [15]. Therefore, although isolation of the bone marrow is an invasive procedure as compared to the adipose tissue, BMSCs should still be an appropriate choice for those seed cells used on bladder tissue regeneration, due to the fact that these cells can be conveniently obtained and expanded, with little inherent immunogenicity, multilineage differentiation potential, and abundant paracrine activity [16].

Previous studies have shown that the intramuscular infusion of BMSCs into detrusor is safe and effective for the treatment of DCP, but in fact, low engraftment of transplanted BMSCs are commonly observed [17, 18]. As the low survival and proliferation of transplanted BMSCs limit the efficacy of this approach, strategies to enhance engraftment and proangiogenic effect of BMSCs might be crucial for greater efficacy in BMSC therapy [19, 20].

In response to the challenges briefly outlined above, genetic modification of BMSCs has been developed for decades and has made great progress in MSCs therapy. Integrin-linked kinase (ILK) is widely expressed on cells [21]. Some studies have proved that ILK and its downstream effector protein pAKT are major in the process of stem cell proliferation, migration, and angiogenesis in the transplanted region [22, 23]. Meanwhile, ILK can act as a pro-survival factor and play a role in protecting cells from high glucose-associated apoptosis and oxidative stress [24, 25]. Overexpression of ILK can promote proliferation and vascular differentiation of stem cells and improve muscular function [26]. We have previously found that overexpression of ILK enhances the regeneration of native smooth muscle cells and improves muscle contractile function [27]. However, little is known about the paracrine effects of ILK gene-modified BMSCs on DCP and related mechanism till now.

In this study, we aimed to investigate whether the treatment of DCP with ILK gene-modified BMSCs enhanced stem cell engraftment, induced vascular formation, relieved ischemia in the transplanted region, and promoted bladder function recovery.

## Materials and methods

### Animals

Male Sprague-Dawley (SD) rats were purchased from the Medical Experimental Animal Center of PLA Nanjing Military (Nanjing, China) and were housed at the Laboratory Animal Research Center of Nanjing Medical University on a 12-h light/12-h dark cycle and with free access to water and food.

### Isolation and culture of BMSCs

BMSCs were collected from the thighbones and shinbones of 4- to 6-week-old Sprague-Dawley rats under sterile conditions. The bone marrow was seeded on cell culture flask using DMEM/F-12 culture medium (Hyclone, USA), supplemented by 10% fetal bovine serum (Gibco, USA) and 1% penicillin-streptomycin mixture (Gibco, USA), and incubated at 37 °C with 5% CO_2_. After 3 days of incubation, the medium was changed and replaced twice a week to eliminate nonadherent cells. When cells reached 80–90% confluence, they were digested with 0.05% trypsin-EDTA (Hyclone, USA) and subcultured at a ratio of 1:2. BMSCs from passages 3–5 were used in all experiments. Meanwhile, the identification of BMSCs was performed as described by our previous study [27], such as cell surface markers, Oil-Red-O staining, and von Kossa staining.

### Establishment of ILK gene-modified BMSCs

Construction of recombinant adenovirus overexpression ILK was performed as described by our previous work [27]. Briefly, the AdMax adenovirus packaging system (Microbix, Canada) was used to insert the integrin-linked kinase/murine cytomegalovirus (ILK-MCMV) fragments into the vector. The adenovirus vector (Ad) titer was determined by the median tissue culture infectious dose, and a titer of 1.7 × 10^11^ pfu/mL was estimated. The optimal multiplicity of infection (MOI) value was determined as 100. The BMSCs were transfected with the ILK gene sequence as the Ad-ILK-BMSC group, the BMSCs were transfected with negative control sequence as Ad-null-BMSC group, and the blank group was not treated at all as BMSC group. After 72 h of transfection, cells were collected and used for follow-up experiments.

### Expression of ILK gene and its related genes

After infection, the expression levels of ILK mRNA and protein were detected by quantitative real-time polymerase chain reaction (qRT-PCR) and western blot. Meanwhile, expression levels of ILK-related genes (AKT, GSK-3β, and CDH1) were also detected through these methods.

### Cell proliferation assay

The number of viable cells in proliferation was assessed using the Cell Counting Kit-8 (CCK-8) assay. The cells were seeded at a density of 10,000 cells per well in 96-well plates and were serum-starved for a certain time (24, 48, 72, 96, 120, 144, and 168 h, respectively) with 100 μl DMEM/F-12 medium. At the indicated time points, the medium in each well was replaced with 100 μl mixed DMEM/F-12 medium containing 10% CCK-8 solution according to the manufacturer’s instructions (Dojindo, Japan), and the cells were incubated at 37 °C for 3 h in a humidified 5% CO_2_ atmosphere. Then, the light absorbance at 450 nm was read using a microplate reader.

### Cell adhesion assay

The protocol of cell adhesion analysis was carried out as described previously [28]. Briefly, BMSCs were stained with Hoechst solution by incubation at 37 °C for 30 min, and cells were plated into 12-well and 96-well plates. After incubation at 37 °C for 1 h, nonadherent Hoechst-labeled cells were removed by careful washing, and PBS was added to each well.

Finally, cell nuclei were stained with Hoechst in PBS for total cell count, and cells in 12-well plates were observed using a Fluoview microscope (Olympus) at the first hour. Subsequently, the fluorescence intensity of each well of 96-well plates was measured using a fluorescein filter set for the following 4 h. The total fluorescence intensity of plated cells was obtained by omitting the wash steps. The percentage of adherent cells by the total corrected fluorescence of cells added to each well and multiplying by 100%.

### Quantitative reverse transcription-polymerase chain reaction

Total RNA was isolated using TRIzol kit (Invitrogen, USA) according to the manufacturer’s instructions. Reverse transcription reaction was conducted using a PrimeScript RT reagent kit (TaKaRa Bio, Japan) as instructed by the manufacturer. cDNA was produced from the total RNA using a FastStart Universal SYBR Green Master (Roche, Germany) and detected on an ABI 7500 Fast Real-Time PCR Instrument (Life Technologies, USA). PCR reactions were performed in triplicate. The specificity of the amplified products was determined by melting-peak analysis. Quantification of each gene of interest (GSK-3β, CDH1, ILK, and AKT) was normalized against the housekeeping gene β-actin analyzed using the 2^-△△CT^ method. Each experiment was performed 3 times. The sequence information for all the primers in this study is listed in Table 1.

**Table 1:** Sequences of primers for the quantitative real-time polymerase chain reaction

Gene symbol	Forward primer (5′-3′)	Rcverse primer (5′-3′)
GSK-3β	GACTAAGGTCTTCCGACCCC	TTAGCATCTGACGCTGCTGT
CDH1	AGCTACCCCAGGACACCCAA	GCAACGCAATCAGAGTCAACG
ILK	TTTGCAGTGCTTCTGTGGGAA	CTACTTGTCCTGCATCTTCTC
AKT	ACTCATTCCAGACCCACGAC	AGCCCGAAGTCCGTTATCTT
β-actin	CCACCATGTACCCAGGCATT	AGGGTGTAAAACGCAGCTCA

### Enzyme-linked immunosorbent assay (ELISA)

BMSCs were incubated in DMEM/F-12 medium containing 10% fetal bovine serum at 37 °C in a humidified 5% CO_2_ atmosphere until they became confluent. Then, the cells were washed twice with phosphate-buffered saline (PBS) and replenished with a serum-free culture medium. The supernatants were collected from the different experimental conditions at culture medium exchange at 72 h and stored at − 80 °C until assayed. After centrifuged at 1500 rpm for 10 min, concentrations of the cytokines including basic fibroblast growth factor (bFGF) and stromal cell-derived factor-1 (SDF-1) were determined in the incubation media with specific quantitative ELISA (Rat bFGF ELISA Kit; Sigma, USA) (Rat SDF-1 ELISA Kit; Sigma, USA). The manufacturers’ protocols for the different kits were followed carefully. Fresh DMEM/F-12 medium was used as a control for the baseline level of cytokines in the culture. The corresponding protein standards, as positive controls, and standard curves were run for each assay.

### Preparation of diabetic rat model

A diabetic rat model was performed using streptozotocin (STZ) described by our previous study [27]. Briefly, SD male rats (76 in total, age 10–12 weeks) were fasted for 16 h before diabetes induction. STZ (Sigma, USA) dissolved in 0.1 ml/L citrate buffer (PH 4.5) was injected intraperitoneally into 66 randomly selected rats (experimental groups) at a dose of 60 mg/kg. The remaining 10 rats (normal control group) received vehicle injection (ice-cold 0.1 mol/L citrate-phosphate buffer [pH 4.2]). After 24 h, 7 days or 10 days, random fasting blood glucose levels of all 66 rats (experimental group) were > 15 mmol/L, which was considered as stable diabetes [29].

### Experimental design

**Fig. 1:**
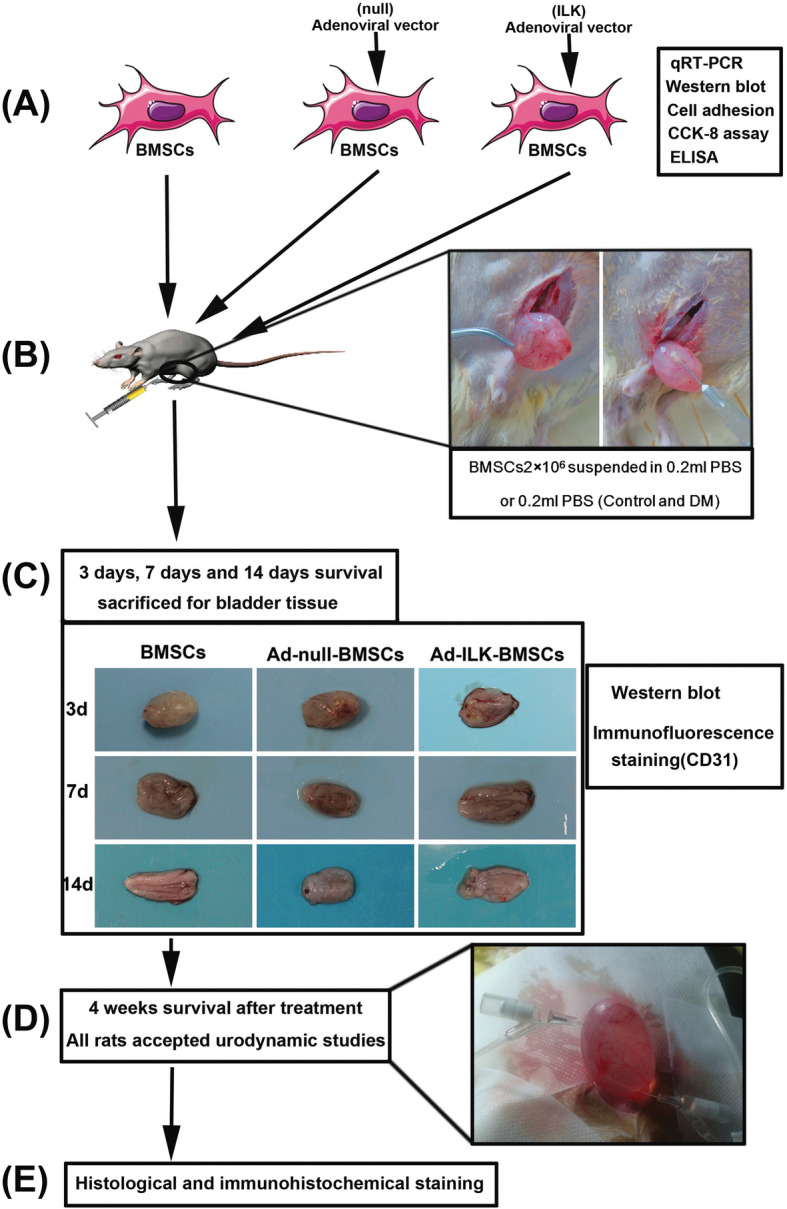
Schematic diagram of experimental design. **a** BMSCs were collected and infected with adenoviral vector carrying the ILK gene sequence. The expression levels of ILK and its downstream protein were detected using quantitative reverse transcription-polymerase chain reaction (qRT-PCR), western blot, and ELISA. Cell adhesion assay and CCK-8 assay were also conducted. **b** Rats in DM group and control group (*n* = 10 per group) accepted PBS injection, and the other three experimental groups accepted pure BMSCs or gene-modified BMSCs (*n* = 16 per group). **c** After cell injection, diabetic rats in experimental groups (BMSC group, Ad-null-BMSC group, and Ad-ILK-BMSC group) (2 rats per group each time) were sacrificed for the bladder tissue on the third day, 7th day, and 14th day, respectively. The angiogenesis in the bladder wall was evaluated using western blot and immunofluorescence staining. **d** At 4 weeks after treatment, all rats in five groups accepted urodynamic studies to evaluate bladder function. **e** After urodynamic studies, the bladders were dissected and used for histological and immunohistochemical staining

To investigate whether ILK gene-modified BMSC treatment could promote angiogenesis and bladder tissue restoration in DCP, we conducted the following experiments, including cell transplantation, detection of the changes in secreted cytokines, observation of the process of angiogenesis in tissue, and bladder function evaluation (Fig. 1).

During the 14 weeks after the injection of STZ, totally 8 rats were lost (mainly 10th to 12th week) which were thought to result from infection, malnutrition, ketoacidosis, and multiple organ failure. Then, the remaining diabetic rats were further divided into the following groups: sham control (control group, *n* = 10); DCP model alone (DM group, *n* = 10); DCP rats intravenously treated with BMSCs (BMSC group, *n* = 16); DCP rats accepted adenoviral vector-infected BMSCs (Ad-null-BMSC group, *n* = 16) and DCP rats accepted ILK adenoviral vector-infected BMSCs (Ad-ILK-BMSC group, *n* = 16). Cell transplantation was performed on the rats in the experimental groups 14 weeks after the establishment of diabetes [30]. Briefly, rats were anesthetized with ether, and a midline incision was made starting at the symphysis and proceeding 2–3 cm cephalad. The peritoneum was entered after the incision of the rectus fascia and separating the rectus muscles. Then, the bladder was well exposed, and 0.2 ml of PBS or BMSC suspension solution (1 × 10^7^ cell/mL) was directly injected into the detrusor layer of the bladder at 5 points (0.04 mL/point) (Fig. 1b). The injection was done from the serosal layer, and the abdomen was closed in a routine manner.

To trance the internal engraftment and proangiogenic effect of BMSCs in the transplanted region of the pathological bladder, protein expression levels of E-cadherin (CDH1), von Willebrand factor (vWF), and vascular endothelial growth factor-A (VEGF-A) were detected by western blot in different phrases of the BMSC treatment. Briefly, diabetic rats in experimental groups (BMSC group, Ad-null-BMSC group, and Ad-ILK-BMSC group) (2 rats per group each time) were sacrificed for the bladder tissue on the third day, 7th day, and 14th day, respectively, after injection of cells by an overdose of 10% chloral hydrate. Meanwhile, the evaluation of angiogenesis in the bladder tissue was made via immunofluorescence staining with CD31.

### Western blot analysis

The total protein of the bladder tissue was extracted using whole-cell lysis assay (Roche, Germany). The protein concentrations of the lysate were determined using a BCA protein assay kit (Pierce Bio-Technology, USA) according to the manufacturer’s instruction.

Fifty micrograms of the sample protein was subjected to sodium dodecyl sulfate (SDS)-10% polyacrylamide gel (SDS-PAGE) analysis and transferred onto a polycinylidenefluoride membrane (Merck Millipore, USA). After blocking nonspecific binding, the membranes were incubated with primary antibody dilutions overnight at 4 °C, followed by 1 h incubation with HRP-conjugated secondary antibody at 37 °C. The immunoreactivity was detected by enhanced chemiluminescence (Amersham, USA) and captured on X-ray film. Each experiment was performed independently 3 times.

### Immunofluorescence staining

The bladder tissue separated from rats in each group was detected by immunofluorescence staining with CD31. All sections were cut on a cryostat and stored free-floating in PBS. Sections were stained overnight at 4 °C with rabbit anti-CD31 (Bioworld Technology, USA) antibody in PBS with 0.2% Triton X-100. Following washing in PBS for 5 min, sections were incubated with fluorescent-conjugated secondary antibodies (Bioworld Technology, USA). Sections were then mounted on slides, dried at room temperature, and covered with Prolong antifade kit (Invitrogen, USA). Confocal images were captured with a Fluoview microscope (Olympus). Image software was used for quantification by using the background subtraction function.

### Bladder function evaluation

The bladder function of all rats was evaluated on the 4th week after the transplantation of BMSCs by the investigators who were blinded to the experimental design. The evaluations were made through urodynamic studies, which were performed just prior to harvesting the urinary bladders.

Briefly, rats were anesthetized with ether, and a vertical abdominal incision was made to expose the bladder. A MedLabs Diagnostics (Jenkintown, USA) pressure transducer catheter was inserted into the bladder to perform cystometry and was filled with physiologic saline at an infusion rate of 0.5 mL/min through another catheter connected to a venous pump. Real-time changes of intravesical pressure were recorded by MedLabs software. Bladder capacity was determined when leakage per urethra or spontaneous voiding occurred. Then, residual volumes were measured after urination. Bladder capacity and vesical pressure were recorded.

### Histological and immunohistochemical staining

The rats were sacrificed after urodynamic studies by an overdose of 10% chloral hydrate. The rat bladders were fixed by perfusion with 4% paraformaldehyde solution. Paraffin sections were dewaxed using xylene and gradient ethanol. Three percent of hydrogen peroxide was used to block the endogenous peroxidase activity. Sections were then incubated sequentially with the antibody (Ki67, 1:500; vWF, 1:500) (polyclonal; Sigma, USA), polymer helper, and poly-HRP anti-mouse IgG. The emissions obtained were developed with diaminobenzidine. Then, the sections were counterstained with hematoxylin, dehydrated in ascending alcohol series, and examined under a light microscope (Olympus) after closing them with neutral balsam. Conventional hematoxylin and eosin (HE) staining and TUNNEL staining were also used on the sections for morphological evaluation of the bladder tissues. The images were acquired using fluorescent microscopy (Leica).

For quantification of the positive cells in the penumbra cortex, three sections from each sample were examined. The positive cells were counted from five randomly selected fields in each section.

### Statistical analysis

All the experimental data were presented as mean ± standard deviation and were analyzed using SPSS 22.0 software. Comparisons of different groups were conducted using independent sample *t* test, while for multiple specimens, statistical analyses were performed using analysis of variance (ANOVA) method. *P* < 0.05 was considered to be statistically significant.

## Results

### The effect of ILK overexpression on rat BMSCs

**Fig. 2:**
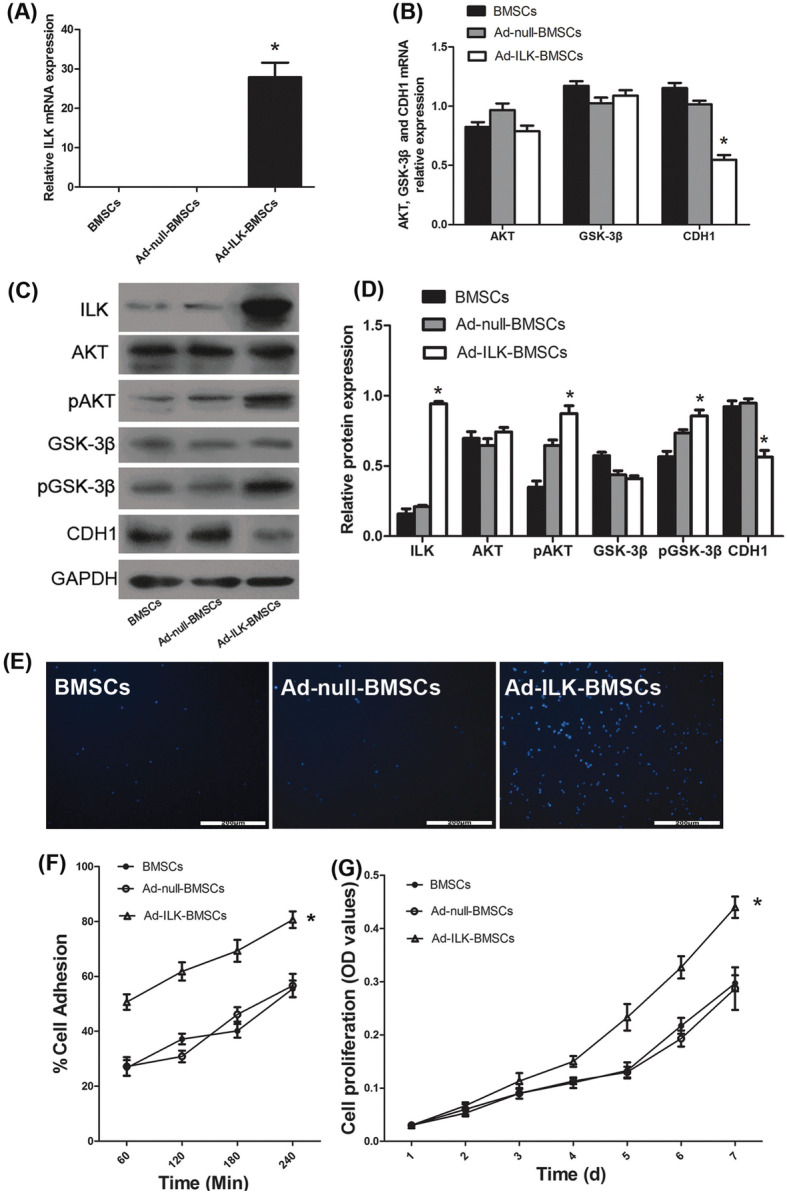
Overexpression of ILK on BMSCs and its effect on proliferation and adhesion of BMSCs. **a** The mRNA expression of ILK was determined using qRT-PCR. *P < 0.05 versus BMSCs and Ad-null-BMSC group. **b** The mRNA expression of ILK downstream protein AKT, GSK-3β, and CDH1 was determined using qRT-PCR. *P < 0.05 versus BMSCs and Ad-null-BMSC group. **c** The protein expression of ILK and its downstream protein AKT, pAKT, GSK-3β, pGSK-3β, and CDH1 by western blot in the BMSCs. **d** A statistical chart of the relative optical density of ILK, AKT, pAKT, GSK-3β, pGSK-3β, and CDH1 in each group. *P < 0.05 versus BMSCs and Ad-null-BMSC group. **e** Adherent cells stained with Hoechst were observed using a Fluoview microscope in cell adhesion assay. **f** A statistical chart reveals the percentage of adherent cells by the total corrected fluorescence of the total cells in each group. *P < 0.05 versus BMSCs and Ad-null-BMSC group. **g** The proliferation level of cells in each group was determined using CCK-8 assay for 7 days. *P < 0.05 versus BMSCs and Ad-null-BMSC group

To investigate the potential role of ILK, we obtained the rat ILK gene sequence and prepared adenovirus to overexpress ILK in BMSCs. After transfection with adenovirus for 72 h, the results of qRT-PCR and western blot showed that the mRNA and protein expression levels of ILK in the Ad-ILK-BMSC group were significantly higher than that in the control group (BMSCs) and Ad-null-BMSC group (Fig. 2a, c, and d). These results demonstrated that ILK was successfully overexpressed in BMSCs. Meanwhile, the expression levels of pAKT and pGSK-3β, but not total AKT and GSK-3β, were significantly upregulated in the Ad-ILK-BMSC group compared to the control and Ad-null-BMSC group. Nevertheless, the expression of CDH1 was inhibited in the Ad-ILK-BMSC group (Fig. 2b–d).

### The effect of ILK on proliferation and adhesion of BMSCs in vitro

As ILK can significantly affect the proliferation and adhesion of cells [31], a series of experiments were conducted to further investigate the biological functions of ILK on BMSCs. Firstly, CCK-8 results demonstrated that the proliferation of the Ad-ILK-BMSC group was significantly elevated (Fig. 2g). Then, cell adhesion assay was performed to investigate the cell adhesion capacity. The results showed that the Ad-ILK-BMSC group had a higher cell adhesion rate compared with BMSCs and Ad-null-BMSC group (Fig. 2e, f). Meanwhile, there was no significant difference in cell proliferation and adhesion between the two control groups. Additionally, western blot analysis confirmed these effects, as this overexpression of ILK in BMSCs significantly increased the phosphorylation of AKT and decreased the expression level of CDH1, subsequently increased the phosphorylation of their common downstream effector protein GSK-3β (Fig. 2c, d). This implies that ILK may improve the survival of BMSCs via ILK/AKT/GSK-3β pathway or ILK/CDH1/GSK-3β pathway [32].

### Results of rat model establishment

After 66 rats were intraperitoneally injected with STZ, fasting blood glucose of all rats was > 15 mmol/L. Eight rats were lost during the molding process, and the other 58 rats were successfully induced into DCP models. Then, the rats which were still alive after model establishment were dived into four groups as described previously. All diabetic rats showed sustained elevated blood glucose levels and weight loss before BMSC injection which were consistent with the characteristics of diabetes (Table 2).

**Table 2:** The general information of rats in each group before cells treatment (*X* ± *S*)

Time point	Control	DM	BMSCs	Ad-null-BMSCs	Ad-ILK-BMSCs
Body weight (g)					
Before modeling	189 ± 11.7	178 ± 12.5	171 ± 8.6	184 ± 10.9	176 ± 9.6
10th week	394 ± 18.1	167 ± 20.4	163 ± 15.9	164 ± 13.7	159 ± 18.5
14th week	503 ± 37.2	162 ± 26.8	160 ± 27.8	162 ± 19.1	157 ± 24.1
Blood glucose level (mM)					
First week	4.7 ± 0.68	22.5 ± 3.8	22.9 ± 4.9	21.8 ± 4.1	23.6 ± 3.6
14th week	5.1 ± 0.41	24.1 ± 4.2	23.8 ± 3.3	23.1 ± 4.7	22.8 ± 4.8

*S* standard deviation, *X* X-bar

The BMSCs were injected into the detrusor layer of the bladder by intraabdominal injection, and the process was shown in Fig. 1c. The general information of the number of rats in different sections was shown in Table 3.

**Table 3:** The number of rats in different sections

		All rats (*n* = 76)				
Initial groups		Control	Diabetic rats model			
Number of rats		10	58 (8 rats were lost before cell transplantation)			
The experimental groups		Control	DM	BMSCs	Ad-null-BMSCs	Ad-ILK-BMSCs
Number of rats		10	10	16	16	16
Sacrificed for the bladder tissue after cell transplantation	Third day			2	2	2
	7th day			2	2	2
	14th day			2	2	2
	4 weeks	10	10	10	10	10

### The effect of ILK gene-modified BMSCs on bladder function recovery

**Fig. 3:**
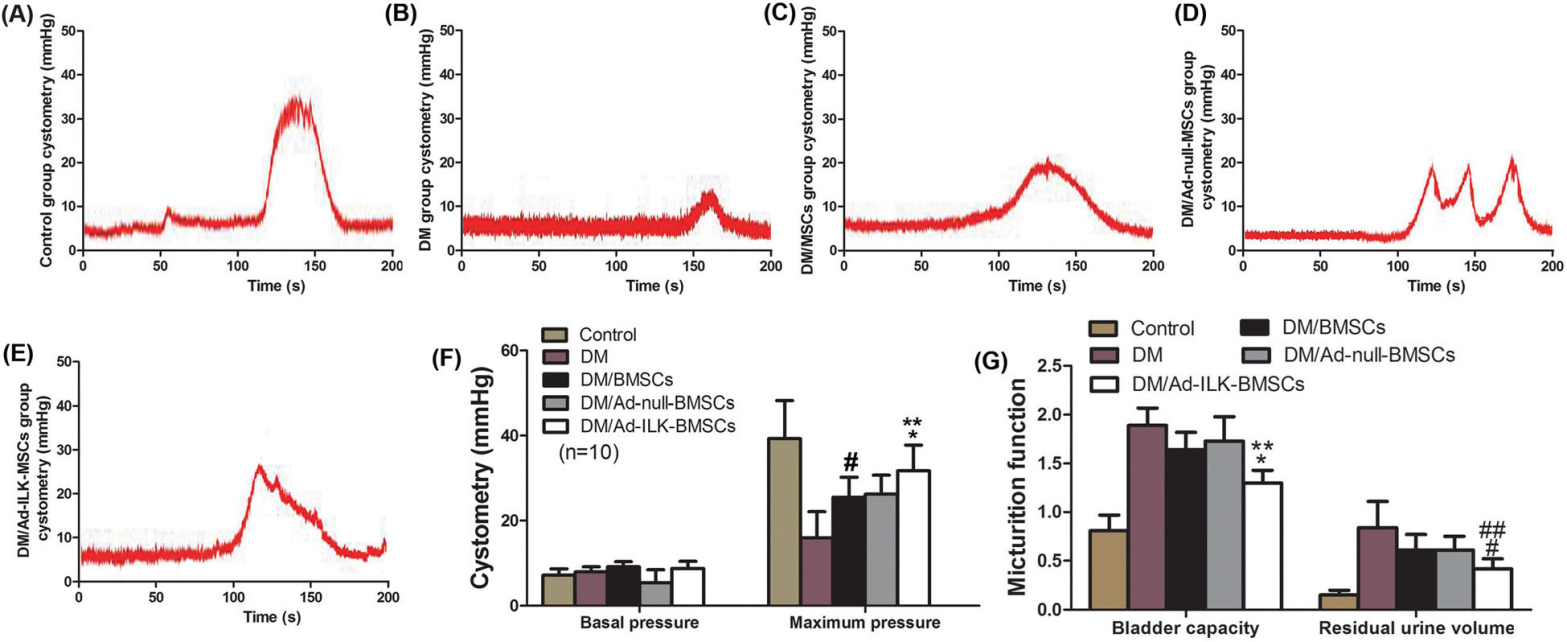
Cystometry recordings illustrated bladder function recovery in experimental group rats. **a**–**e** Cystometry variables of the **a** sham control, **b** DM, c DM + BMSCs, **d** DM + Ad-null-BMSCs, and **e** DM + Ad-ILK-BMSC groups. **f** A statistical chart of cystometric parameters (basal pressure and maximum pressure) in each group. *P < 0.05 versus BMSCs and Ad-null-BMSCs. **P < 0.05 versus DM. #*P* < 0.05 versus DM. **g** A statistical chart of bladder micturition function (bladder capacity and residual urine volume) in each group. *P < 0.05 versus DM. **P < 0.05 versus BMSCs and Ad-null-BMSCs. #*P* < 0.05 versus DM. ##*P* < 0.05 versus BMSCs and Ad-null-BMSCs

To determine whether ILK gene-modified BMSCs improved bladder function recovery, urodynamic studies were performed at 4 weeks after treatment. There were comparable cystometric parameters among the five groups. Representative cystometrograms (Fig. 3a–e) revealed that the bladder contractile function and micturition threshold volume in the cell-transplanted groups were significantly better than those in the DM group. Meanwhile, the most significant improvement of the bladder function was observed in the Ad-ILK-BMSC group (Fig. 3f, g).

### The proangiogenic effect of ILK gene-modified BMSCs

According to recent literatures, ILK can promote the proangiogenic activity of MSCs via their paracrine effect [33, 34]. Thus, the proangiogenic ability of ILK gene-modified BMSCs was evaluated in vitro and in vivo.

**Fig. 4:**
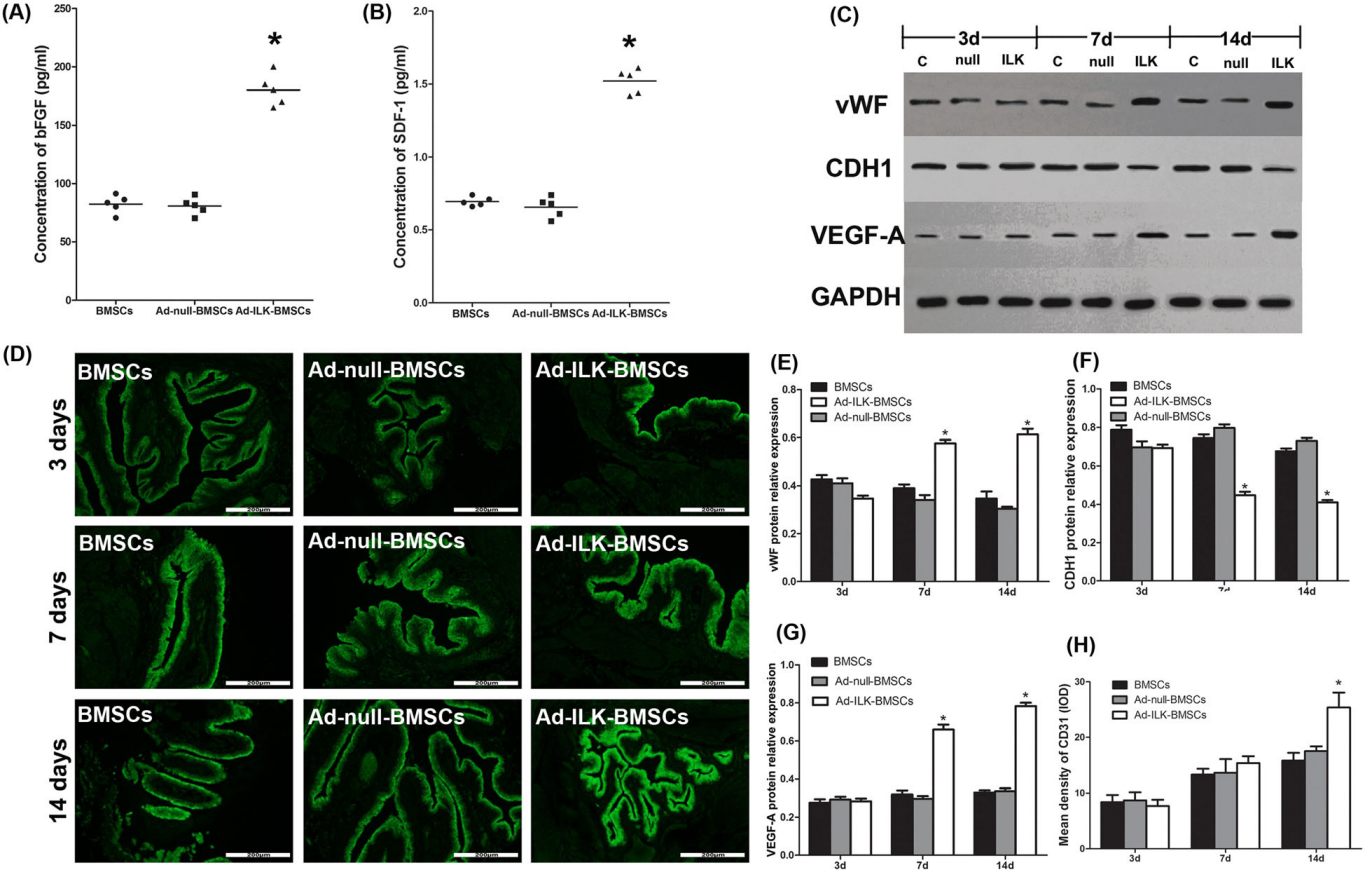
The dynamic changes of angiogenesis in the bladder tissue at different stages of the treatment. **a–b** Graphical representation of culture supernatant bFGF and SDF-1 concentrations determined by ELISA for each group in vitro. The concentration of bFGF and SDF-1 in the Ad-ILK-BMSC group was significantly higher (*P < 0.05) than those in the BMSCs and Ad-null-BMSC group. **c** The protein expression of CDH1 (one indicator of migration), VEGF-A (one indicator of angiogenesis), and vWF (one indicator of vascular endothelial cell marker) by western blot in the bladder wall of each group at different stages of the treatment. **d** The expression of CD31 (one indicator of vascular endothelial cell marker) by immunofluorescence staining in the bladder wall of each group at different stages of the treatment. **e–g** A statistical chart of the relative optical density of CDH1, VEGF-A, and vWF in each group. *P < 0.05 versus BMSCs and Ad-null BMSC group. **h** A statistical chart of the mean density of CD31 in each group. *P < 0.05 versus BMSCs and Ad-null BMSC group

In vitro, ELISA results demonstrated that the expression levels of bFGF (one indicator of angiogenesis) and SDF-1 (one indicator of migration) were significantly upregulated after ILK overexpression (Fig. 4a, b).

In vivo, after the transplantation of cells, a total of 18 rats of experimental groups were sacrificed for bladder tissue on the third day, 7th day, and 14th day of treatment, respectively, ahead of schedule (2 rats per group each time) (Fig. 1c). Then, western blot was performed to investigate the dynamic changes of the expression levels of CDH1 (one indicator of migration), VEGF-A (one indicator of angiogenesis), and vWF (one indicator of vascular endothelial cell marker) in the process of BMSC treatment. The results of western blot showed no significant difference in the expression of vWF, CDH1, and VEGF-A among the three experimental groups on the third day. On the 7th day, the expression of vWF and VEGF-A in the Ad-ILK-BMSC group was significantly higher than that in the BMSC group and Ad-null-BMSC group, but the expression of CDH1 was decreased in the Ad-ILK-BMSC group. On the 14th day, the positive effect on the expression on vWF and VEGF-A became more obvious compared with before (Fig. 4c, e–g). These results demonstrated that the expression of vWF and VEGF-A were successfully upregulated on the 7th day after BMSC transplantation approximately; meanwhile, the expression of CDH1 was decreased. To investigate the proangiogenic effect of ILK on BMCSs in vivo, immunofluorescence staining with CD31 (one indicator of vascular endothelial cell marker) was performed on the bladder tissue separated from rats accepted BMSC treatment. The results showed that the percentage of the vascular area significantly increased in the Ad-ILK-BMSC group compared with the BMSCs and Ad-null-BMSC group on the 14th day (Fig. 4d, h).

**Fig. 5:**
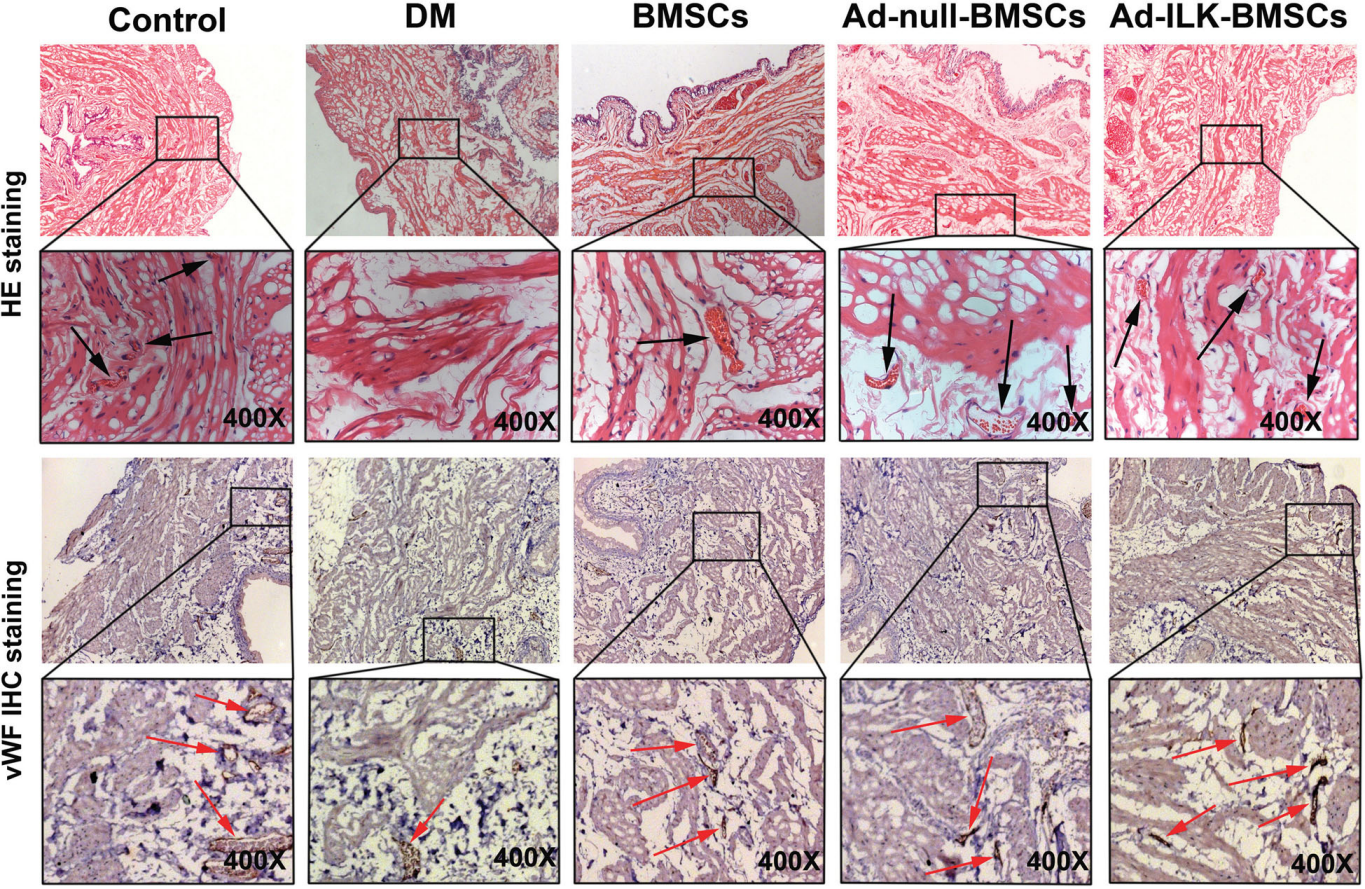
ILK promotes angiogenesis in vivo. HE and vWF IHC staining of corresponding bladder tissues from all five groups are shown. Arrows represent open lumens

Then, after 4 weeks of treatment, all rats (*n* = 10, per group, five groups) were sacrificed for bladder tissue. The results of immunohistochemistry (IHC) staining showed that the expression level of vWF in the Ad-ILK-BMSC group was stronger than that in the DM, BMSCs, and Ad-null-BMSC group (Fig. 5). The results of HE and IHC revealed that the Ad-ILK-BMSC group had more blood vessels feeding into a new organization. These findings mean that ILK gene-modified BMSCs can effectively promote the process of angiogenesis in the transplanted region via their enhanced paracrine effects.

### The ILK gene-modified BMSCs affect hyperglycemia-induced apoptosis in vivo

**Fig. 6:**
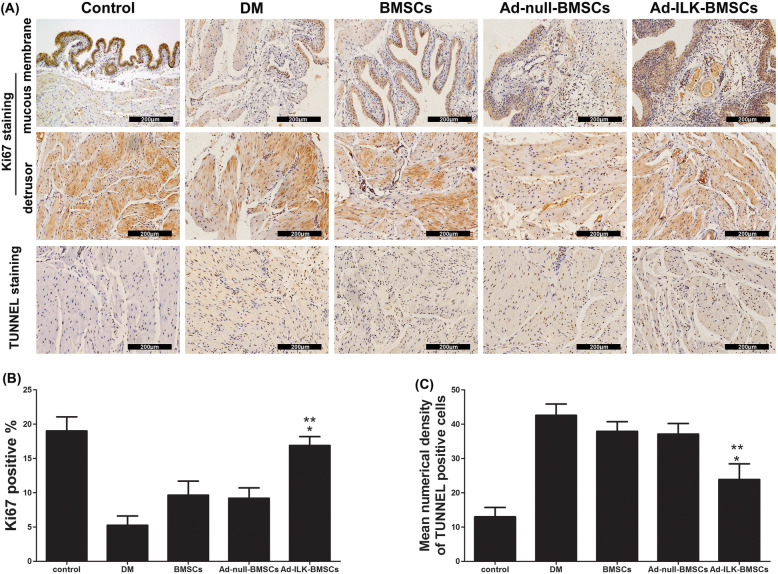
Effects of ILK overexpression on cell apoptosis. **a** Ki67 IHC staining and TUNNEL staining were performed to evaluate cell apoptosis in the bladder tissues from each group. **b** A statistical chart of the percentage of Ki67-positive cells showed the proliferation level of cells in each group. *P < 0.05 versus BMSCs and Ad-null BMSC group. **P < 0.05 versus DM group. **c** A statistical chart of mean numerical density of TUNNEL-positive cells showed apoptosis level of cells in each group. *P < 0.05 versus BMSCs and Ad-null BMSC group. **P < 0.05 versus DM group

Considering the unclear effects of ILK on cell proliferation and angiogenesis in vivo, we suspect that ILK has a protective effect on hyperglycemia-induced apoptosis. After 4 weeks of BMSC treatment, we detected the change of cell biological function. IHC by Ki67 staining and TUNNEL staining in paraffin sections was performed to investigate the proliferation and apoptosis of cells in the bladder tissue from all five groups. The results of TUNNEL staining showed that, after cells transplanted treatment, least TUNNEL immunopositive cells which form the bladder tissue were observed in the evaluation of the Ad-ILK-BMSC group. And immunopositivity of cross-sections from the DM group was remarkable (Fig. 6a, b). Meanwhile, the results of Ki67 staining showed that, compared with BMSCs and Ad-null-BMSC group, the proportion of Ki67 positivity among the bladder tissue is higher in the Ad-ILK-BMSC group, indicating high cell proliferation (Fig. 6a, c). Accordingly, these results demonstrated that ILK gene-modified BMSCs may provide a protective effect for the ischemia bladder tissues against massive apoptosis caused by hyperglycemia.

**Fig. 7:**
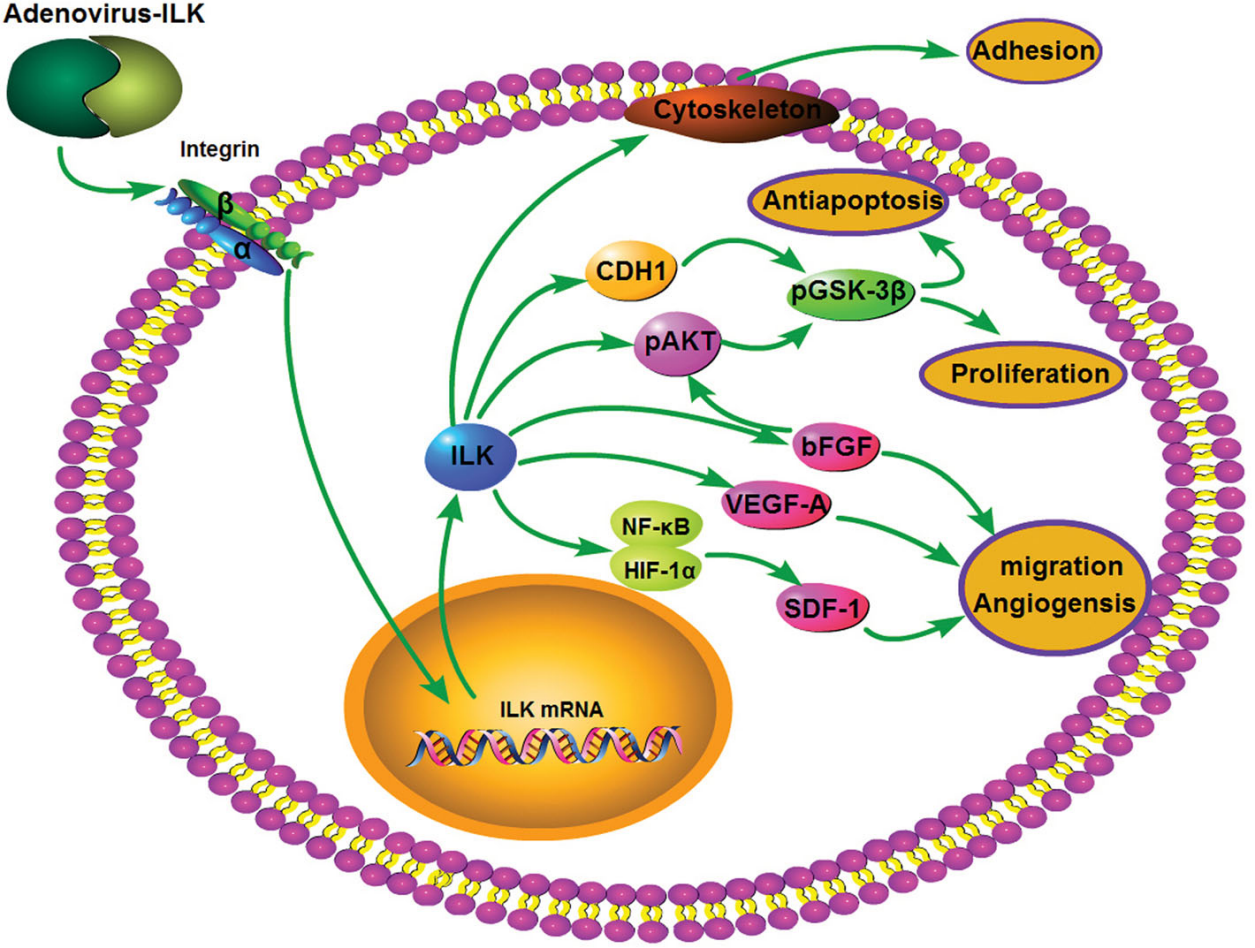
A schematic representation of possible mechanism for ILK-mediated proliferation, adhesion, angiogenesis, and antiapoptosis in BMSCs

These data indicated that overexpression of ILK effectively improved the proliferation and adhesion ability of BMSCs, as well as provided a protective effect on hyperglycemia-induced apoptosis in the bladder tissue. The paracrine effects of ILK gene-modified BMSCs were enhanced, presented with the increased expression of bFGF, VEGF-A, and SDF-1. Then, the angiogenesis in the tissue was promoted through the positive effect of these trophic factors, which led to the vascular formation and relieved bladder ischemia in diabetic rats (Fig. 7).

## Discussion

In this study, we demonstrated that the overexpression of ILK significantly increased phosphorylation of AKT and GSK-3β and decreased expression of CDH1, and enhanced engraftment of BMSCs in the transplanted region than the BMSC therapy alone via the activation of AKT pathway. Furthermore, we also found that the overexpression of ILK promoted angiogenesis and antiapoptosis in the BMSC transplanted treatment and enhanced function outcome of DCP. These effects were related to the greater secretion of bFGF, SDF-1, and VEGF-A of BMSCs when infected with adenovirus-ILK.

Currently, despite numerous studies on bladder tissue regeneration using MSCs, the cellular mechanisms underlying regeneration remain poorly understood. It is still unknown how undifferentiated MSCs enhance bladder tissue regeneration. One possible mechanism includes direct differentiation of implanted MSCs into desired cell types under signals from the surrounding microenvironment of the bladder tissue. Meanwhile, MSCs may stay undifferentiated and release growth factors, which act as a feeder layer rich in trophic factors that trigger the migration of native cells from the surrounding tissues. At this point, a recent study concerning bladder tissue regeneration revealed that differentiation plays a minor role in the final regeneration effect mediated by MSCs [13]. Furthermore, BMSCs are commonly used in differentiating into SMCs and urothelium in the case of bladder tissue regeneration [35]. Therefore, BMSCs became the ideal seed cells in our study.

Despite numerous MSC-based therapies have been reported to improve function outcome of underactive bladder in rat models [36–38], a major limitation to the use of MSCs in clinical applications is their poor viability at the site of injury due to the hyperglycemia-induced ischemic microenvironment and to anoikis driven by the loss of cell adhesion. Therefore, to improve the long-term survival of the transplanted MSCs, strategies to regulate apoptotic signaling and enhance cell proliferation and adhesion have been developed, such as genetic modifications [39]. Recently, a series of studies have demonstrated that the treatment of genetically modified MSCs showed improvement of function recovery than MSC monotherapy [40, 41]. For example, ILK gene therapy has been reported to improve cardiac remodeling and function in rats after myocardial infarction and was associated with increased angiogenesis, reduced apoptosis, and increased cardiomyocyte proliferation [42].

The gene functions of ILK are primarily driven by phosphorylating AKT, GSK-3β, and many other substrates [43]. And ILK has been reported to be implicated in cell growth and survival by protection from apoptosis through modulation of these downstream targets [44]. Meanwhile, ILK-dependent pathway can also activate β-catenin/LEF-mediated gene transcription and downregulate CDH1 protein expression, lead to increased phosphorylation of GSK-3β, and promote cell proliferation [45]. ILK has also been reported to act as a pseudokinase to transduce a non-catalytic signal to promote cytoskeleton reassembly and dynamic cell adhesion [46]. As shown in Fig. 2, in vitro section, the overexpression of ILK triggered the increased phosphorylation of AKT and GSK-3β and decreased expression of CDH1, leading to the improvement of the proliferation of BMSCs. These in vitro data revealed that overexpression of ILK could effectively enhance the proliferation and adhesion of BMSCs, which may improve the long-term survival of the transplanted MSCs in vivo [39].

The main mechanisms of MSCs in the reconstitution of bladder dysfunction are attributed to the cell migration, differentiation, and paracrine effects. Recent studies have demonstrated that ILK can strongly induce angiogenesis with increased expression of VEGF and SDF-1 [47, 48]. SDF-1 has been reported to contribute to the migration of stem cells into ischemia tissue [49]. It has been reported that ILK overexpression in cells can result in SDF-1 upregulation through dual control by nuclear factor-kappaB (NF-κB) and hypoxia-inducible factor-1alpha (HIF-1α) [47]. Furthermore, ILK also has been proved to increase cell migration through the downregulation of the expression level of CDH1 [50]. Meanwhile, several studies have revealed that bFGF and VEGF are identified as downstream of ILK, which are important molecules involved in migration and proliferation of vascular endothelial cells and smooth muscle cells, and are viewed as multipotent angiogenic stimuli that are important for tissue regeneration [48, 51, 52]. In this study, our results showed that ILK gene-modified BMSCs significantly increased SDF-1, bFGF, and VEGF-A expression levels and decreased the CDH1 expression level compared with BMSC treatment, subsequently promoted the vascular remodeling in hyperglycemia-induced ischemia of the bladder tissue. Our study initially confirmed this improvement of vascular formation induced by these cytokines on the 14th day of ILK gene-modified BMSC treatment (Fig. 4).

Then, we hypothesized that the mechanism of such ILK gene therapy also concerns the enhancement of migration and paracrine effects of BMSCs, which results in the improvement of several pivotal physiological processes of angiogenesis.

To maximize the therapeutic effect of MSCs, many researchers have attempted to optimize the methodology aspects, including the source of MSCs, delivery method, transplant timing, and dosage [53, 54]. However, these optimizations have showed limited improvement in the poor survival of the graft due to the ischemia microenvironment. Thus far, new strategy, such as genetic modification, has been developed and has showed promising outcomes in MSC therapy. Recently, increasing evidence indicates that the genetically modified MSCs always have a better outcome than wild-type MSCs due to the expression of the function genes after transfection [55]. The functions of the gene targeted for MSC modification are classified as follows: firstly, enhance proliferation and antiapoptosis capacity; secondly, improve homing and migration ability; and thirdly, increase paracrine effects or transdifferentiation capacity.

In this study, our results showed that the ILK gene-modified BMSC treatment of DCP significantly enhanced the process of angiogenesis in the tissue and improved function outcome than BMSC monotherapy. On the other hand, in the ischemia region of the bladder tissue, the ILK gene-modified BMSC treatment significantly increased the number of the blood vessels, which could improve the harsh ischemia microenvironment. These results suggest that the therapeutic effect of ILK gene-modified BMSCs on angiogenesis may relate to the enhancement of migration and paracrine effects in the ischemia region.

However, there are some limitations to this study. Due to the positive evaluation of angiogenesis, antiapoptosis, and bladder tissue regeneration, we hypothesized that the engraftment of BMSCs was enhanced in vivo, but except for the in vitro detection of cell viability by CCK-8 and cell adhesion capability, there is no in vivo data to directly evaluate the engraftment of BMSCs. Therefore, further investigation concerning dynamic cell adhesion, proliferation, migration, and differentiation in the transplanted region during postoperative period would allow us to gain further insight into the underlying mechanisms and exactly improve MSC treatment.

## Conclusions

In summary, we demonstrate that the ILK gene-modified BMSC treatment significantly enhances the engraftment and migration of BMSCs,and increases paracrine effects to potentiate angiogenesis and bladder function recovery after DCP. These results suggest that the gene-modified treatment might be a beneficial strategy for DCP in a clinical setting.

## Data Availability

Data sharing is not applicable to this article because no datasets were generated or analyzed during the current study.

## Abbreviations

DCP: Diabetic cystopathy
MSCs: Mesenchymal stem cells
ILK: Integrin-linked kinase
BMSCs: Bone marrow-derived stem cells
STZ: Streptozotocin
qRT-PCR: Quantitative real-time polymerase chain reaction
AKT: Protein kinase B
GSK-3β: Glycogen synthase kinase-3β
CDH1: E-cadherin
CCK-8: Cell counting kit-8
VEGF: Vascular endothelial growth factor
bFGF: Basic fibroblast growth factor
SDF-1: Stromal cell-derived factor-1
vWF: Von Willebrand factor
HE: Hematoxylin and eosin
IHC: Immunohistochemistry
